# Calcium-Phosphate Subchondroplasty Provides Durable 24-Month Pain Relief and >90% Surgery-free Survivorship for Tibiofemoral Bone Marrow Lesions in Mild-To-Moderate Knee Osteoarthritis: A Prospective Multicenter Study

**DOI:** 10.1177/19476035261476205

**Published:** 2026-08-31

**Authors:** Giuseppe Anzillotti, Elizaveta Kon, Pietro Conte, Peter Angele, Pieter Emans, Joan Minguell-Monyart, Jennifer Woodell-May, Miguel Correa-Tapia, Berardo Di Matteo

**Affiliations:** 1 9268 IRCCS Humanitas Research Hospital, Rozzano, Milan, Italy; 2 Department of Biomedical Sciences, Humanitas University, Pieve Emanuele, Milan, Italy; 3 Department of Trauma and Reconstructive Surgery, 39070 University Hospital Regensburg, Regensburg, Germany; 4 Sporthopaedicum Regensburg/Straubing, Regensburg, Germany; 5 Joint Preserving Clinic, Department of Orthopedic Surgery, 199236 Maastricht University Medical Center, Maastricht, The Netherlands; 6 Department of Orthopaedics & Traumatology Surgery, 16810 Vall d’Hebron University Hospital, Barcelona, Spain; 7 Universidad Autònoma de Barcelona, Barcelona, Spain; 8 433896 Zimmer Biomet, Warsaw, IN, USA; 9 Clinical Affairs, 36418 Zimmer Biomet, Winterthur, Switzerland

**Keywords:** subchondroplasty, knee, osteoarthritis, subchondral bone, calcium phosphate

## Abstract

**Purpose:**

To report 24-month clinical outcomes, surgery-free survivorship, and radiological variations after Calcium-Phosphate (CaP) Subchondroplasty (SCP) procedures in patients with symptomatic knee bone marrow lesions (BMLs) in the context of mild-to-moderate knee osteoarthritis (OA).

**Methods:**

Adults with tibiofemoral BMLs (Kellgren–Lawrence [KL] 1–3) underwent CaP SCP using a standardized protocol. Prespecified outcomes were KOOS (primary: KOOS Pain), EQ-5D-5L, and pain NRS at baseline, 1, 3, 6, 12, and 24 months; surgery-free survivorship (Kaplan–Meier); and structural status by KL and MOAKS (available-case imaging). Analyses compared time points with paired tests and 95% CIs.

**Results:**

Ninety-two knees were treated. KOOS Pain improved from 45.9 ± 14.3 preoperatively to 81.3 ± 18.1 at 24 months (*p* < .0001). All other KOOS subscales improved from baseline to 24 months (all *p* < .0001) and pain NRS decreased from 6.8 ± 1.5 to 3.3 ± 2.6 (*p* < .0001). Kaplan–Meier surgery-free survivorship was 97.7% (95% CI, 90.9–99.4) at 12 months and 91.5% (95% CI, 82.9–95.8) at 24 months; 7/92 knees (7.6%) underwent reoperation. KL distributions showed no significant change versus baseline at 12 months (*p* = .5089) or 24 months (*p* = .9801). MOAKS improved at 12 months (*p* = .0435) but not at 24 months (*p* = .2517) in the available MRI subset. No serious device-related adverse events were observed.

**Conclusion:**

In BML-positive knees with KL 1–3 OA, SCP yielded large and durable improvements in pain, function, and health-related quality of life through 24 months, with >90% surgery-free survivorship and radiographic stability.

## Graphical abstract

**Graphical Abstract:**
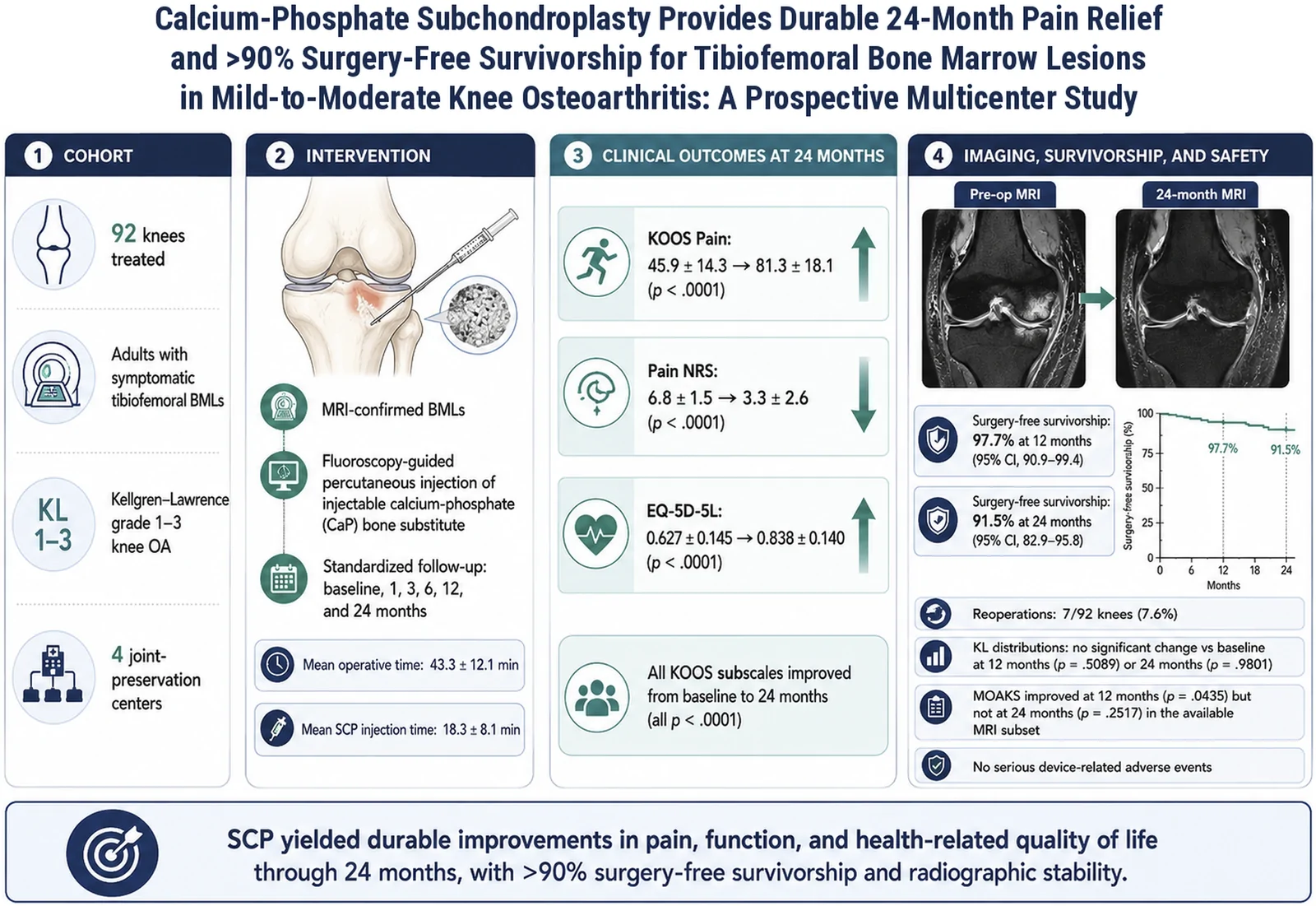

## Introduction

Osteoarthritis (OA) of the knee is now well recognized as a disease of the whole joint, in which alterations of cartilage are coupled with subchondral bone, synovium and menisci interacting as a mechanical and biological unit.^1,2^ Across this complex interaction, subchondral bone is not merely a bystander but an active contributor to symptoms and structural deterioration.^3-6^ Among subchondral changes, MRI-visible bone marrow lesions (BMLs) represent a dynamic, clinically meaningful radiological signal that integrates histological alteration of the subchondral bone (i.e. micro-fracture, fibrosis, edema, cyst formation, and vascular and nerve ingrowth), consistently linked to pain and progression.^7,8^ Longitudinal imaging studies have shown that BMLs fluctuate over time and that their presence and severity are able to accurately predict subsequent cartilage loss, worsening of symptoms, and even the need for joint replacement.^7,9,10^ Therapeutic strategies targeting the subchondral compartment have therefore gained traction, aiming to interrupt bone–cartilage crosstalk that perpetuates nociception and structural failure.^11-15^ Subchondroplasty® (SCP)—the percutaneous, fluoroscopy-guided injection of an injectable calcium phosphate (CaP) bone substitute into the BMLs area—seeks to provide immediate mechanical support while creating an osteoconductive scaffold that remodels to bone.^16-18^ Early prospective and retrospective clinical series and systematic reviews reported improvements in pain and function, with heterogeneous methodology and limited radiological data.^19-23^ Despite growing adoption, important knowledge gaps remain. Medium-term durability (>12 months), radiographic/MRI trajectories under standardized scoring, and surgery-free survivorship have been inconsistently reported. A recent multicenter case series suggested favorable clinical outcomes and a 5-year survivorship free of arthroplasty,^24^ although overall data still remains limited. The aim of the present multicenter prospective clinical study is to determine the 24-month clinical effectiveness and safety of subchondroplasty for symptomatic tibiofemoral bone marrow lesions in mild-to-moderate knee osteoarthritis. It was hypothesized that, in carefully selected knees with KL 1–3 OA and persistent tibiofemoral BMLs, SCP would produce clinically meaningful and durable improvements in pain, function, and health-related quality of life through 24 months, with high surgery-free survivorship and no significant radiographic progression.

## Materials and Methods

### Study Design

This was a prospective, multicenter cohort study conducted at four joint-preservation centers (Humanitas Research Hospital, Rozzano, Italy; Universitätsklinikum Regensburg, Germany; Maastricht University Medical Centre+, the Netherlands; and Vall d’Hebron University Hospital, Barcelona, Spain). The protocol was approved by the local Ethics Committees at all sites (Ethikkommission der Universität Regensburg (Germany), reference number 17-744-101; the Comitato Etico Indipendente dell’Istituto Clinico Humanitas (Italy), authorization number 1963; Medisch-Ethische Toetsingscommissie (METC) azM/UM at Maastricht UMC+ (Netherlands), reference numbers NL62987.068.17 and METC171093; and Comité de Ética de Investigación con Medicamentos (CEIm) of Hospital Universitari Vall d’Hebron (Spain), code PR(ATR)469/2017), and the study was registered on ClinicalTrials.gov (NCT03430219). Written informed consent was obtained from all participants. The present report extends outcomes of the previously published 12-month cohort to 24 months.

### Participants

Adults (≥18 years) with radiologically confirmed knee OA and at least one MRI-confirmed BML in the tibiofemoral compartment were eligible. Key inclusion criteria were Kellgren–Lawrence (K–L) grade 1–3; persistent knee pain for ≥3 months with KOOS-Pain <70; coronal alignment within ±6°; and failure of nonoperative care (i.e. physical therapy, joint unloading). Principal exclusions were local or systemic infection; osteonecrosis on MRI; systemic inflammatory disease; metabolic bone disease; patellofemoral OA; K–L grade 0 or 4; and prior surgery on the index knee within 12 months. Screening required a prior MRI showing a BML; after enrollment, standardized radiographs were performed to assess the K–L grade and a pre-operative MRI (within 30 days from the scheduled surgical procedure) was performed to confirm BML persistence and plan the subchondroplasty treatment. For eligibility, a BML was defined as an ill-defined area of increased signal intensity within the subchondral and epiphyseal trabecular bone on fluid-sensitive, fat-suppressed sequences (proton-density– or T2-weighted fat-saturated/STIR), with corresponding low-to-intermediate signal on T1-weighted images, consistent with the edema-like signal of osteoarthritis. Only BMLs in direct subchondral contiguity with the tibiofemoral articular surface were included, whereas purely cyst-like lesions and lesions attributable to acute trauma, osteonecrosis, or a subchondral insufficiency fracture were excluded. Lesions were classified by location (medial/lateral femoral condyle, medial/lateral tibial plateau) on the baseline MRI; lesion extent was graded with the MOAKS bone-marrow-lesion size sub-score (0–3 per subregion).^25^

### Intervention (Subchondroplasty® Procedure)

All procedures were performed by experienced surgeons using fluoroscopic guidance. After spinal anesthesia, cannulas were advanced percutaneously into the target BML(s) based on preoperative MRI planning. Accurate intralesional placement was ensured by combining preoperative planning with intraoperative biplanar fluoroscopy: the location, depth, and trajectory of each lesion were defined on the preoperative MRI relative to fixed bony landmarks (joint line, cortical margins, intercondylar notch), and true anteroposterior and lateral fluoroscopic views were used to confirm that the cannula tip reached the subchondral target corresponding to the MRI-identified lesion. Correct intraosseous positioning was further verified by the characteristic resistance on advancement and by visualization of controlled intratrabecular spread of the calcium-phosphate material under real-time fluoroscopy, without extra-osseous or intra-articular leakage; the cannula was repositioned when needed to optimize fill. An injectable calcium-phosphate (CaP) bone substitute (AccuFill®, Zimmer Biomet) was prepared per kit instructions and delivered intraosseously to fill, but not overpressurize, the lesion. Cannulas were kept in place during setting (∼10–12 minutes), then removed. Arthroscopy was performed after cannula removal to detect and clear any intra-articular extrusion; no additional concomitant procedures were allowed. Postoperatively, patients followed a standardized pathway: analgesia and thromboprophylaxis per protocol, partial weightbearing for 1-week, progressive weightbearing by ∼2 weeks, immediate range-of-motion, and progressive strengthening while avoiding high-impact activity for 3 months.

### Outcomes and Follow-Up Schedule

Clinical assessments were collected at baseline and at 1, 3, 6, 12, and 24 months and included the Knee Injury and Osteoarthritis Outcome Score (KOOS; all subscales), EuroQol-5 Dimensions-5 Levels (EQ-5D-5L), and a Numeric Rating Scale (NRS) for pain. All instruments were administered in each participant’s native language using previously validated translations and have demonstrated acceptable reliability and validity in knee-osteoarthritis populations.^26-30^ The 24-month analysis focused on durability of clinical benefit and surgery-free survivorship. K–L grading was obtained by analyzing standard X-rays. MRI assessments were performed through the MRI Osteoarthritis Knee Score (MOAKS) to characterize BMLs: MRI was performed at baseline and at 12 months’ follow-up; where a 24-month MRI was available as part of routine care, MOAKS was scored and analyzed exploratorily.

### Definitions of Failure

Failure was defined as the need of any subsequent surgical procedure on the index knee due to persisting or relapsing symptoms (e.g., conversion to unicompartmental or total knee arthroplasty, cartilage procedures, joint distraction).

### Statistical Analysis

The primary endpoint was the change in KOOS Pain score from baseline to 12 months after the SCP. Secondary endpoints included change from baseline in the remaining KOOS subscales, EQ-5D, NRS pain, healthcare utilization, subject global satisfaction, imaging variables, adverse events, and incidence and time to reoperation or revision, assessed at 1, 3, 6, 12, and 24 months. Continuous variables were reported as mean ± standard deviation, with sample size, range, median, and 95% confidence intervals, and categorical variables as counts and percentages . For longitudinal clinical outcomes, the report presented comparisons of each follow-up visit versus baseline (“preop”), reported as “p-value Comparing Change From Preop”, using either T-test (Satterthwaite) or T-test (Pooled) depending on the endpoint 1. A one-way ANOVA was reported for the numeric global satisfaction measures across visits and for comparisons of 12-month KOOS scores by lesion location. Additional exploratory between-group t-tests (Pooled) at 12 months were presented for gender and number of lesions. Surgical survivorship was evaluated using the Kaplan-Meier method, with survival estimates and 95% confidence intervals reported at 12 and 24 months 1. No multiplicity adjustment, censoring rules, attrition assumption, minimum required sample size, or explicit significance level was specified in the report. A p of 0.05 defined statistical significance. No formal a priori sample-size or power calculation was performed for the present study; the analysed sample comprised all consecutively enrolled eligible knees treated at the four participating centres during the recruitment period, so that the achieved sample size reflects case availability and feasibility rather than a pre-specified statistical target. The 24-month evaluation is a pre-planned extension of this prospective cohort, whose 12-month outcomes have been reported previously.

## Results

### Patient Flow and Baseline Characteristics

Ninety-two consecutive knees were enrolled and treated. A summary of baseline demographics and lesion features is provided in Table 1. In brief, 57.6% were male, the index knee was left in 54.3%, and most cases (63.0 %) presented a single BML; the most frequent lesion site was the medial femoral condyle (51.1%), followed by the medial tibial plateau (25.0%). Regarding MOAKS score at baseline, approximately two-thirds of patients (62.2%) exhibited none or mild structural abnormalities (Grades 0–1), whereas 36.0% presented with moderate-to-severe findings (Grades 2–3), indicating a mixed severity profile at study entry.

**Table 1.** Characteristics of the Included Patients

​	Value (N = 92)
Age	54 ± 16
BMI	28 ± 4.7
Sex	
Males	53/92 (57.6%)
Females	39/92 (42.4%)
Index knee	
Left	50/92 (54.3%)
Right	42/92 (45.7%)
Kellgren-Lawrence grade	
0	-
1	28
2	41
3	23
4	-
Number of BMLs	
1	58/92 (63%)
2	34/92 (37%)
BML #1 location	
Lateral femoral condyle	9/92 (9.8%)
Lateral tibial plateau	13/92 (14.1%)
Medial femoral condyle	47/92 (51.1%)
Medial tibial plateau	23/92 (25%)
BML #2 location	
Lateral femoral condyle	2/34 (5.9%)
Lateral tibial plateau	3/34 (8.8%)
Medial femoral condyle	4/34 (11.8%)
Medial tibial plateau	25/34 (73.5%)

### Procedural Details and Safety

Mean operative time was 43.3 ± 12.1 minutes; mean SCP injection time was 18.3 ± 8.1 minutes. Mean AccuFill® volume was 3.26 ± 1.00 mL for the primary lesion and 2.58 ± 0.77 mL for the eventual second lesion, whenever present. No device-related serious adverse events were recorded.

### Clinical Outcomes

All clinical scales improved significantly from baseline and improvements were maintained up to 24 months (all *p*< .0001 vs baseline). KOOS Pain increased from 45.9 ± 14.3 preoperatively to 81.3 ± 18.1 at 24 months (mean percent improvement +86.0%). KOOS Symptoms, ADL, Sports, and QoL likewise improved at each time point, with 24-month means of 81.5 ± 20.4 (Symptoms), 80.7 ± 20.5 (ADL), 44.2 ± 31.3 (Sports), and 62.0 ± 27.0 (QoL).

**Figure 1.**
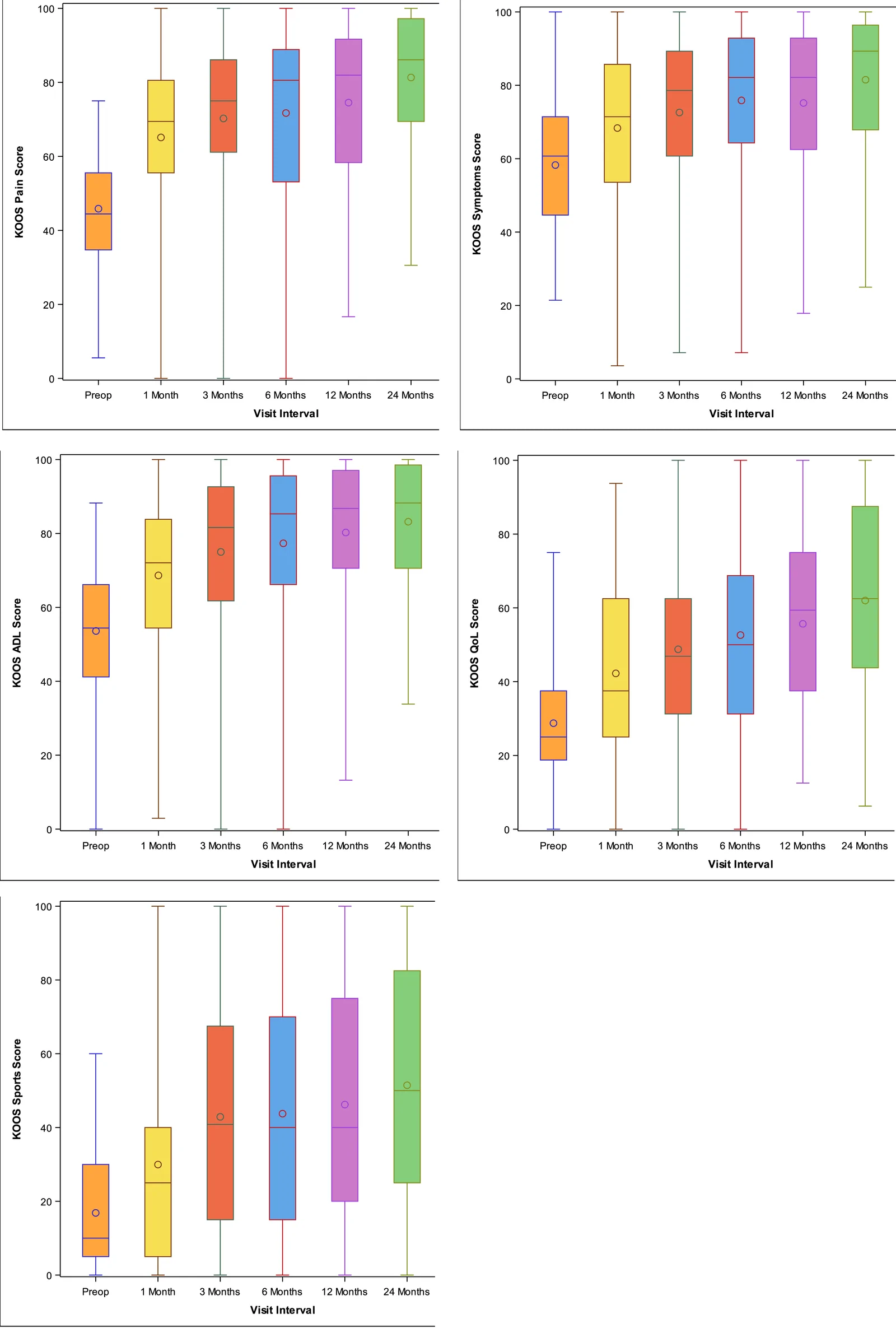
Clinical outcomes assessed (KOOS score) from preoperative timepoint up to 24-month follow-up

**Figure 2.**
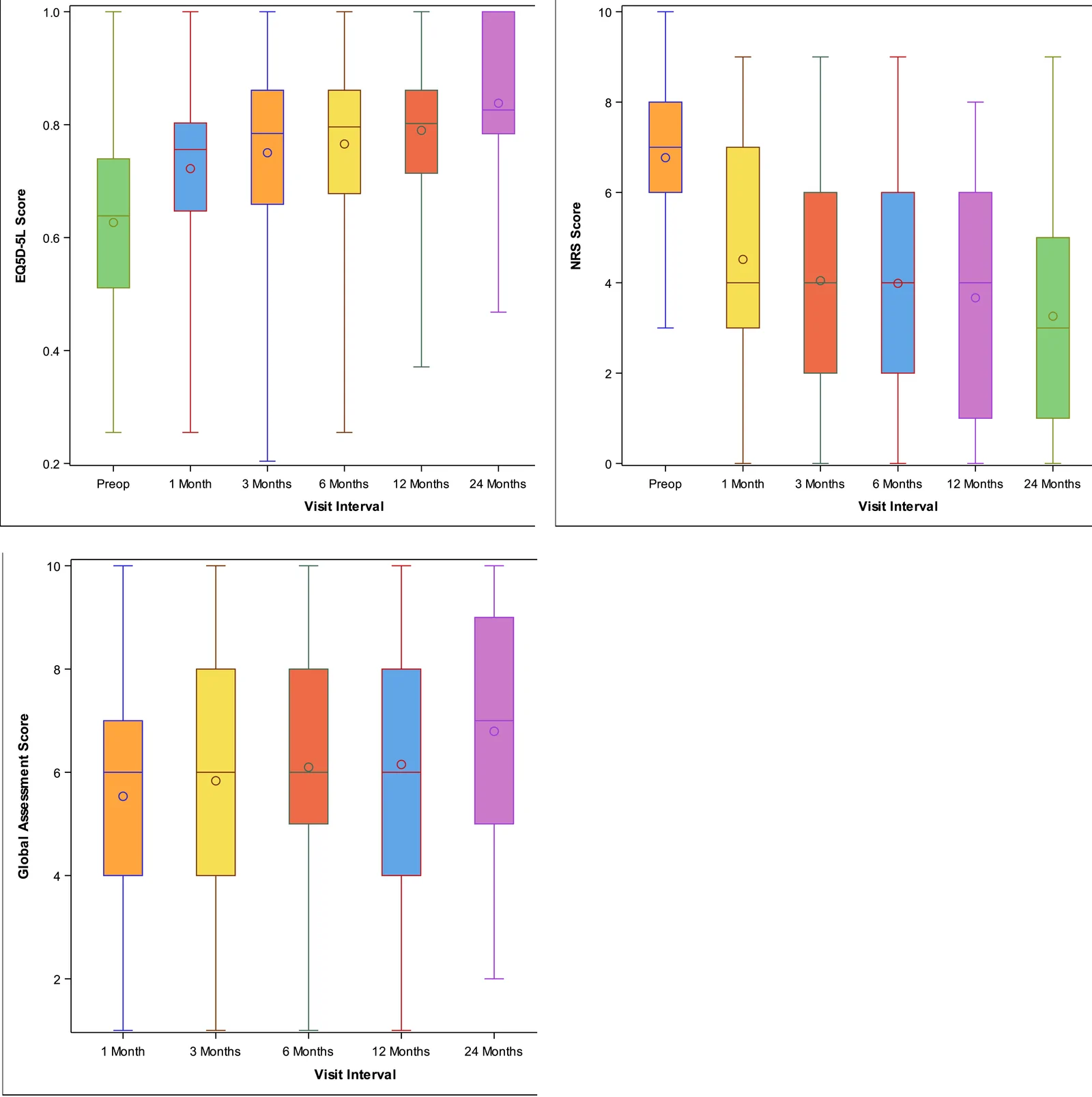
Clinical outcomes assessed (EQ-5D-5L, NRS, Global Assessment score) from preoperative timepoint up to 24-month follow-up

Pain on the NRS decreased from 6.8 ± 1.5 at baseline to 3.3 ± 2.6 at 24 months in the present cohort (*p* < .0001 vs baseline). Health-related quality of life (EQ-5D-5L index) improved from 0.627 ± 0.145 at baseline to 0.838 ± 0.140 at 24 months (both *p* < .0001 vs baseline). Patient-reported global assessment and satisfaction were favorable at 24 months (global assessment 6.8 ± 2.2; satisfaction 7.5 ± 2.5). All the clinical outcomes assessed are shown in Figures 1 and 2.

### Radiographic and MRI Outcomes

Radiographic OA severity by Kellgren–Lawrence grading remained stable over 24 months with no significant shift in distribution (overall *p* = .9801). On MRI, At 12 months, the observed distribution (excluding missing data) suggested a shift toward lower MOAKS grades. Grade 0 remained stable at 35 patients (34.0%), while Grade 1 decreased slightly to 25 patients (24.3%). More pronounced reductions were observed in higher grades: Grade 2 decreased from 21.4% to 13.6%, and Grade 3 from 14.6% to 3.9% (p = .0435). At 24 months, the apparent distribution among evaluable patients indicated further decreases in higher MOAKS grades: Grade 0 was observed in 9 patients (8.7%), Grade 1 in 7 (6.8%), Grade 2 in 4 (3.9%), and Grade 3 in 1 patient (1.0%)(p = .2517). However, the majority of the cohort (78 patients; 75.7%) was missing at this timepoint, with an additional 4 cases (3.9%) classified as unavailable.

**Figure 3.**
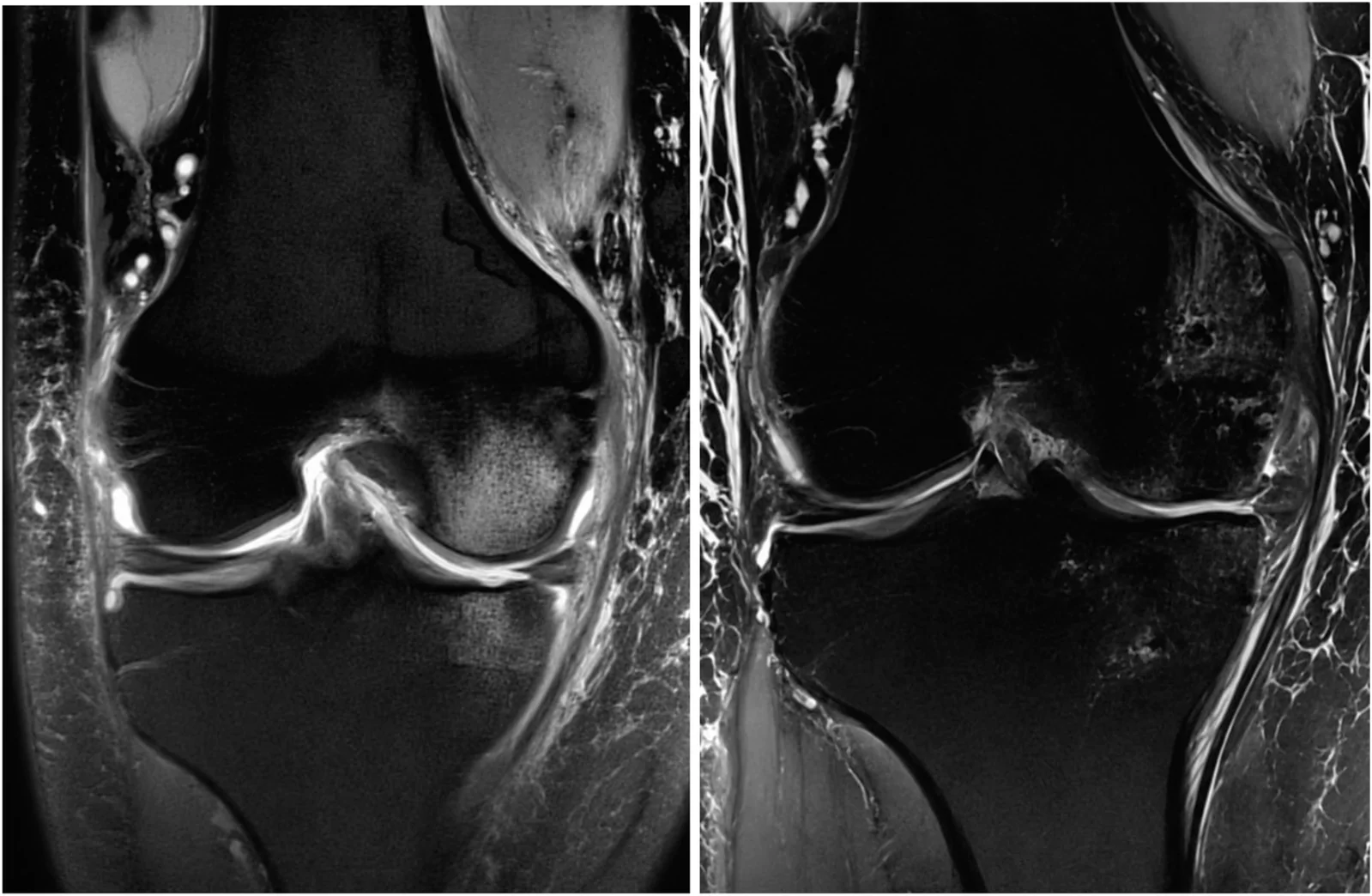
Representative coronal MRI of an enrolled patients undergone subchondroplasty procedure. LEFT) preoperative MRI showing BMLs present at both medial femoral condyle and medial tibial plateau; RIGHT) postoperative imaging, acquired at 24-month follow-up, showing resolution of both the BMLs

A representative example of the BML change before and after subchondroplasty is shown in Figure 3.

### Reoperations and Surgery-free Survivorship

**Figure 4.**
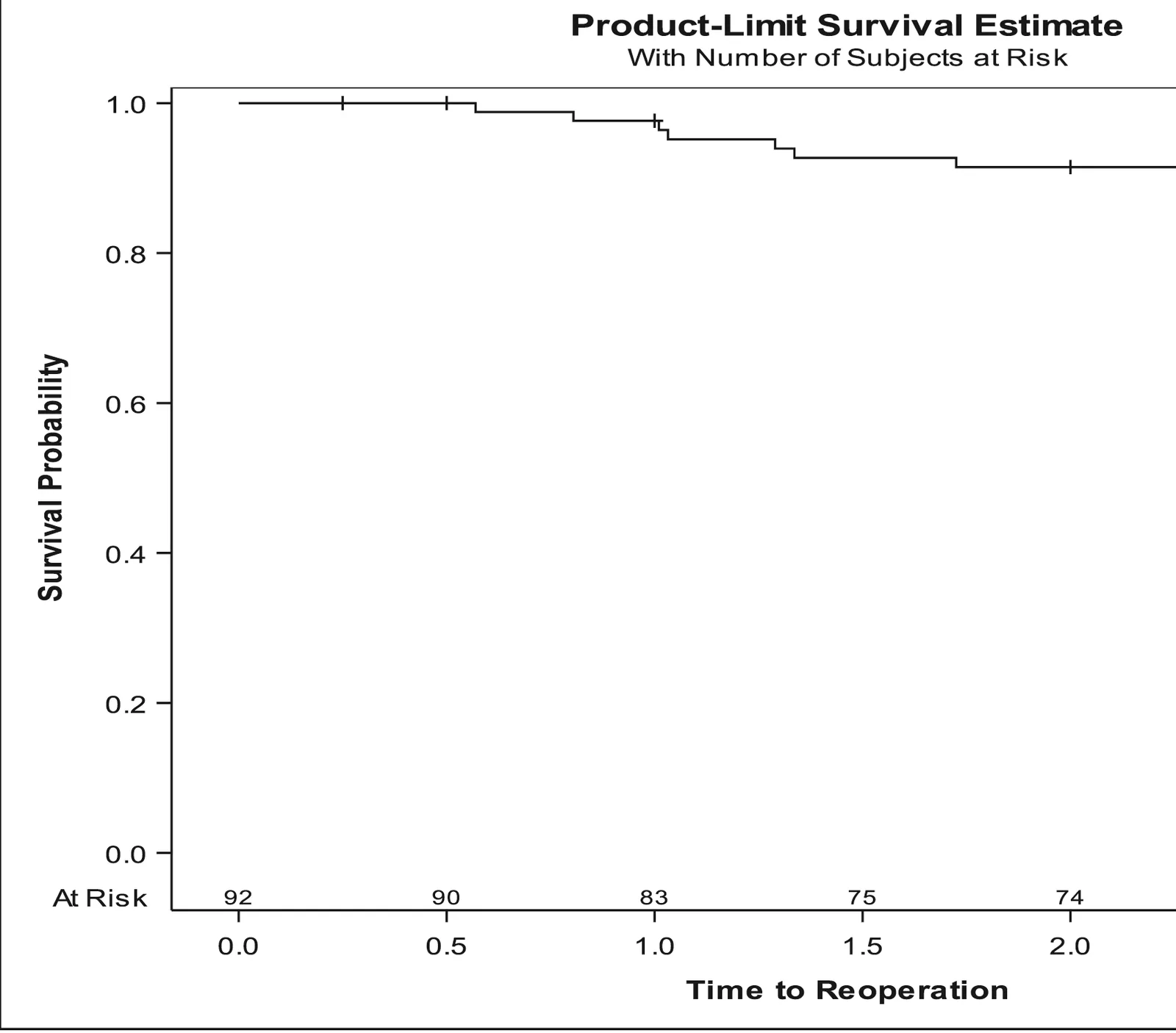
Kaplan-Meier analysis assessing the subchondroplasty survivorship up to 2-year follow-up

Kaplan–Meier analysis of freedom from surgical reintervention on the index knee demonstrated 97.7% (95% CI, 90.9–99.4) survivorship at 12 months and 91.5% (95% CI, 82.9–95.8) at 24 months. Censoring is indicated at last follow-up (Figure 4).

By 24 months, 7 of 92 knees (7.6%) underwent a subsequent surgical procedure on the index knee: conversions included 2 total knee arthroplasties and 1 unicompartmental knee arthroplasty; 1 joint distraction; and 3 cartilage procedures. In 3/7 cases, the reintervention involved the same compartment/BML region treated at the index procedure. No device-related complications necessitating surgery were recorded.

## Discussion

The main finding of this prospective, multicenter trial on patients with mild-to-moderate knee OA and persistent tibiofemoral BMLs is that SCP yielded significant and sustained improvement in pain, function, and health-related quality of life up to 24 months’ evaluation, with an overall surgery-free survivorship of 91.5%. Radiographically, KL grading remained stable, whereas MOAKS score evaluating specifically BMLs showed a decrease from 12 to 24 months in the subset of patients with paired MRIs. The present data extend the previously reported 12-month outcomes in the same cohort and suggest that, in carefully selected BML-positive knees, SCP can provide durable symptom relief without an overt radiographic penalty at two years.^23^

SCP has been increasingly adopted as a joint-preserving option for painful OA associated with BMLs, yet much of the literature consists of single-center case series with heterogeneous indications and variable imaging follow-up. Two systematic reviews concluded that SCP is associated with clinically meaningful pain and function gains, while underscoring the predominance of lower-level evidence and short follow-up.^16,17^ This prospective, multicenter 2-year study corroborates these findings in a cohort of patients who were evaluated with pre-specified clinical outcomes and structured imaging endpoints. Recent studies align with the findings retrieved from the present study. Randelli et al. reported symptomatic improvement after SCP in an arthritic-knee population with BMLs, including follow-up up to 24 months, albeit in a single center case series with mixed imaging availability.^19^ Chua et al. (Level II) likewise found pain relief and functional gains, and Pasqualotto et al. also documented improvement in an early prospective experience.^20,31^ Mid-term survivorship data are emerging. Wood et al. analyzed a large, multi-surgeon database and observed a 5-year survivorship from conversion to arthroplasty of 73% (95% CI 67–79%), identifying older age and grade-4 chondral damage as risk factors for failure.^22^ The 2-year survivorship of 91.5% compares favorably in the near-term and supports the adopted patient-selection strategy focused on KL 1–3 OA. The protocol’s upper limit of KL 3 likely contributed to the favorable clinical trajectory and low conversion rate: conversely, Chatterjee et al. reported poor outcomes for subchondral CaP injection in patients with end-stage disease, thus further strengthening that SCP is not a salvage strategy for extensively degenerated joints.^32^

The present results should also be considered against subchondral decompression techniques that do not involve injection of a bone substitute. Subchondral drilling (core decompression), usually in addition to orthobiologics, of the lesion aims to relieve intraosseous pressure and stimulate a local reparative response, and has been reported to provide pain relief and functional improvement in osteoarthritic and pre-osteoarthritic knees with BMLs.^33-35^ Relative to decompression, calcium-phosphate SCP additionally provides immediate structural support of the weakened subchondral trabeculae and an osteoconductive scaffold that remodels to bone, which may be particularly relevant in the load-bearing tibiofemoral compartment. In the absence of a head-to-head comparison, however, superiority of bone-substitute augmentation over decompression alone cannot be claimed; both approaches share the rationale of treating the symptomatic subchondral lesion, and a randomized comparison of SCP versus subchondral decompression (with and without orthobiologic intraosseous injection) represents the most relevant next step.

Regarding the radiological evolution of BMLs, these findings should be interpreted with caution. Indeed, BMLs are dynamic lesions that can wax and wane over time, which complicates their use as a structural endpoint. The MOAKS improvement registered at 12 months was not confirmed at 24 months, although clinical scores were still favorable. This could be a consequence of the lack of full MRI data at the last follow-up, and the discordance between the satisfactory clinical status and BML relapse may reflect the known fluctuation of BMLs and the multifactorial nature of OA; but also sampling limitations since the size of the trial was calculated on patients’ reported outcomes. Remarkably, no radiographic signal of KL grade worsening was observed up to two years. Future studies with protocolized serial MRI and quantitative BML metrics could clarify structure–symptom coupling after SCP.^3,25^

Moreover, although MRI-detected marrow signal abnormalities in an osteoarthritic knee are often grouped under the umbrella term “BML,” they are heterogeneous, and a true OA-related BML should be distinguished from other entities (e.g., traumatic contusion, inflammatory marrow change, subchondral insufficiency fracture/SONK spectrum, or osteonecrosis) because these differ in natural history and management.^3,7^

In particular, subchondral insufficiency fracture of the knee (SIFK) can mimic an “arthritic BML,” yet represents a stress fracture in which MRI findings such as a subchondral hypointense fracture line and early osteochondral collapse carry important prognostic implications that may not be captured by BML size alone.^36,37^ Even within OA, BML morphology appears heterogeneous (e.g., subchondral versus cyst-like patterns or lesions adjacent to soft-tissue attachments), and emerging work suggests that BML subtypes may relate differently to symptoms and other whole-joint structural features.^38^ Histologic correlation studies further indicate that the classic “edema-like” MRI signal in OA typically reflects a composite of trabecular microdamage, fibrosis/necrosis, and active remodeling rather than true interstitial edema, reinforcing the concept of a mechanically and biologically active osteochondral microenvironment in OA-BMLs.^39-41^ Consistent with this, transcriptomic profiling of OA-BML tissue demonstrates upregulation of pathways linked to osteochondral turnover, angiogenesis, neurogenesis, and inflammation, providing a biological rationale for interventions aimed at modifying the local subchondral lesion milieu.^12,42^

From a mechanistic standpoint, OA-BMLs appear strongly load-related: compartmental overloading has been associated with the presence and longitudinal worsening of BMLs, and both static malalignment and dynamic varus thrust increase the odds of incident or enlarging medial BMLs over time.^43-45^ Meniscal dysfunction is another key mechanical contributor, with meniscal pathology and extrusion predicting incident/enlarging BMLs and structural worsening within the same compartment, supporting the idea that persistent abnormal load transmission can sustain or re-create the subchondral lesion phenotype.^46-48^ In this context, the rationale for SCP in arthritic BMLs extends beyond “filling a lesion,” aiming instead to address focal subchondral microdamage within an overloaded osteochondral unit, while acknowledging that uncorrected mechanical drivers (alignment, meniscal pathology/extrusion, or gait-related thrust) may limit durability and should be considered as potential effect modifiers in future studies. Indirect support for the “treatable target” concept also comes from lesion-directed medical trials: early proof-of-principle data suggested that intravenous zoledronic acid could reduce pain and BML size in selected OA patients, although a larger randomized trial did not demonstrate a benefit on cartilage volume loss and subsequent meta-analytic syntheses have reported inconsistent or absent effects on pain/BML outcomes overall.^49-52^ Finally, imaging biomarker studies indicate that BML regression can track with symptomatic improvement without necessarily translating into reduced concurrent radiographic joint-space narrowing progression, underscoring the need to interpret BML change as supportive (rather than definitive) evidence of disease modification when evaluating subchondral interventions in OA.^53^

In recent years, the subchondral compartment is also being targeted with biologic augmentation approaches (e.g., platelet rich plasma or mesenchymal stem cells), often combined with intra-articular injections. Early prospective data suggest clinical benefit and BML reduction up to 24 months, but heterogeneity of product preparation and protocols and small samples temper conclusions.^54^ Moreover, biologic options targeting the subchondral bone only address the inflammatory aspects of the disease, not the structural side. To this regard, the biologic integration of the AccuFill® combines immediate mechanical support with an osteoconductive scaffold that remodels over time. In vitro and in-vivo studies have shown replacement of injected calcium phosphate by new bone several years after SCP, offering biological rationale for the durable symptom improvement seen in properly selected patients. Nevertheless, these data remain limited to case-level evidence; whether histological integration systematically tracks with clinical response merits further study.^18^

In conclusion, the major strengths of this study rely on its multicentric nature and both radiological and clinical outcomes assessed. The survivorship of the present study compares favorably to mid-term observational data and aligns with systematic reviews indicating that SCP may delay arthroplasty in a subset of patients. It is therefore reasonable to conclude that careful patient selection—considering age, cartilage status, alignment, and compartmental disease—remains essential. Incorporating standardized imaging (e.g., MOAKS) may enhance phenotyping and help in identifying prognostic factors for a correct decision making approach.^16,17,22^

Nonetheless, the present study suffers from several limitations. First, it is a single-arm cohort without a comparator and, therefore, the causal effect of the SCP procedure needs to be confirmed in the setting of a randomized controlled trial against subchondral decompression alone and ortho-biologic intra-osseous injections. Second, the lack of full imaging data at 24 months limits the interpretation of MRI assessment. Third, while multicenter participation improves generalizability, inter-surgeon nuances in decision-making process during the conservative pre-inclusion stage and cannula placement during the procedure may introduce performance variability. Finally, survivorship was defined as freedom from subsequent surgical procedures in the index knee and this does not capture other non-operative interventions occurred during the study period. In addition, although coronal alignment was constrained to ±6°, this report did not formally model the association between the degree of residual malalignment and clinical or survivorship outcomes; given the load-related behaviour of BMLs, alignment should be regarded as a candidate effect modifier in future, adequately powered analyses.

## Conclusion

Subchondroplasty for tibiofemoral BMLs in KL 1–3 osteoarthritic knees produced durable 24-month improvements in pain and function, with 91.5% surgery-free survivorship and no device-related serious adverse events. No radiographic progression of OA progression was observed during the study period; MOAKS improvement at 12 months was not maintained at 24 months in patients with paired MRIs. Overall, these findings support SCP as a safe joint-preserving strategy for knees with persisting BMLs associated to mild-to-moderate OA. Randomized comparative studies with protocolized serial MRI are warranted.

## Data Availability

The data that support the findings of this study are available from the corresponding author upon reasonable request.*
